# Integrative bioinformatics identifies postnatal lead (Pb) exposure disrupts developmental cortical plasticity

**DOI:** 10.1038/s41598-018-34592-4

**Published:** 2018-11-06

**Authors:** Milo R. Smith, Priscilla Yevoo, Masato Sadahiro, Christine Austin, Chitra Amarasiriwardena, Mahmoud Awawda, Manish Arora, Joel T. Dudley, Hirofumi Morishita

**Affiliations:** 1 Department of Neuroscience, Icahn School of Medicine at Mount Sinai, 1 Gustave L Levy Place, New York, NY 10029 USA; 2 Department of Genetics and Genomic Sciences, Icahn School of Medicine at Mount Sinai, 1 Gustave L Levy Place, New York, NY 10029 USA; 3 Department of Psychiatry, Icahn School of Medicine at Mount Sinai, 1 Gustave L Levy Place, New York, NY 10029 USA; 4 Departmnt of Ophthalmology, Icahn School of Medicine at Mount Sinai, 1 Gustave L Levy Place, New York, NY 10029 USA; 5 Department of Environmental Medicine & Public Health, Icahn School of Medicine at Mount Sinai, 1 Gustave L Levy Place, New York, NY 10029 USA; 6 Department of Dentistry, Icahn School of Medicine at Mount Sinai, 1 Gustave L Levy Place, New York, NY 10029 USA; 7 Mindich Child Health and Development Institute, Icahn School of Medicine at Mount Sinai, 1 Gustave L Levy Place, New York, NY 10029 USA; 8 Graduate School of Biomedical Sciences, Icahn School of Medicine at Mount Sinai, 1 Gustave L Levy Place, New York, NY 10029 USA; 9 Institute for Next Generation Healthcare, Icahn School of Medicine at Mount Sinai, 1 Gustave L Levy Place, New York, NY 10029 USA; 10 Friedman Brain Institute, Icahn School of Medicine at Mount Sinai, 1 Gustave L Levy Place, New York, NY 10029 USA

## Abstract

Given that thousands of chemicals released into the environment have the potential capacity to harm neurodevelopment, there is an urgent need to systematically evaluate their toxicity. Neurodevelopment is marked by critical periods of plasticity wherein neural circuits are refined by the environment to optimize behavior and function. If chemicals perturb these critical periods, neurodevelopment can be permanently altered. Focusing on 214 human neurotoxicants, we applied an integrative bioinformatics approach using publically available data to identify dozens of neurotoxicant signatures that disrupt a transcriptional signature of a critical period for brain plasticity. This identified lead (Pb) as a critical period neurotoxicant and we confirmed *in vivo* that Pb partially suppresses critical period plasticity at a time point analogous to exposure associated with autism. This work demonstrates the utility of a novel informatics approach to systematically identify neurotoxicants that disrupt childhood neurodevelopment and can be extended to assess other environmental chemicals.

## Introduction

Of 138 million unique chemical substances (Chemical Abstracts Service; CAS; accessed March 2018: http://support.cas.org/content/chemical-substances) over 84,000 may be commercially produced (excluding pesticides, drugs, cosmetics, and some other substances) and 2,800 are considered high production volume (≥1 million pounds per year) and are likely at elevated levels in the human environment^1^. Given that the vast majority of these chemicals have an unknown, but potential capacity to harm neurodevelopment, there is an urgent need for systematic approaches to identify damaging chemicals. Epidemiological and animal studies have identified specific environmental chemicals that impact prenatal neural events, such as proliferation, migration, differentiation, apoptosis, and gliogenesis. However, knowledge of neurotoxicants disrupting postnatal and childhood periods is less well established, though increasingly observed as an important window of vulnerability^2^.

Childhood neurodevelopment is marked by critical periods of brain plasticity wherein neural circuitry is optimized by the environment to establish normal cognition and behavior^3^. If critical periods are disrupted, development of normal function can be permanently altered and may increase risk for neurodevelopmental disorders such as autism spectrum disorders (ASD)^4,5^. Despite the potential for a deleterious impact on health, the role of environmental chemicals on critical period plasticity has received minimal attention, with only a few disruptors of developmental plasticity identified^6,7^.

Though systematic assessment of the impact by environmental chemicals on health is not yet standard, high-throughput approaches are actively being developed including the S1500 effort led by the U.S. multiagency collaborative, “Toxicology in the 21st Century” (Tox21 program), that aims to screen the transcriptional impact of tens of thousands of drugs in cell lines^8^ as well as independent efforts to screen hundreds of drugs using primary neuronal cultures^9^. Prior to these efforts, high-throughput approaches typically relied on biochemical and cell-based experimental assays using a limited number of gene or protein expression readouts or enzymatic activities. While these assays are important and useful, such assays do not reflect complex *in vivo* neurodevelopmental events. On the other hand, *in vivo* animal assays used in isolation are low-throughput and only appropriate for validation of screening results. Due to these limitations, to our knowledge, no studies have conducted a systematic assessment of environmental chemicals that disrupt complex *in vivo* neurodevelopmental processes such as critical period plasticity. Here, by leveraging the ability of transcriptional signature matching to identify functional and mechanistic relationships^10^ we developed and applied an integrative bioinformatics approach as a systematic screen to identify chemicals that disrupt *in vivo* critical period plasticity. To do so, we computationally matched diverse chemical exposure signatures to an *in vivo* critical period signature and then performed experimental validation *in vivo*. We derived the critical period signature from the primary visual cortex (V1) of the mouse model for ocular dominance plasticity^11^, which has emerged as an indispensable model to dissect the molecular mechanisms subserving developmental plasticity^5^. A well-characterized model^11,12^, ocular dominance plasticity is conserved across mammals including humans, where it peaks during early childhood^13^. This critical period is marked by the capacity of the visual cortex to undergo neural reorganization in response to changes in environmental stimuli, as modeled in the laboratory by depriving one eye of light, which leads to enduring eye-specific loss of visual responsiveness in the visual cortex^12^. Importantly, many of the underlying mechanisms are shared with other brain regions and functions^14^, suggesting that findings derived from the ocular dominance model may be generalizable. By matching transcriptional signatures based on this model to hundreds of disease signatures, we previously showed that an integrative bioinformatics approach is able to identify damaging disease pathways that disrupt plasticity *in vivo*^15^. In the present study, we computationally matched the ocular dominance critical period signature to a subset of hundreds of neurotoxicant signatures among the vast environmental chemical space. Specifically, we focused our analyses on 214 chemicals with established neurotoxic impact on human^16^, since little is known about the impact of these neurotoxicants on critical periods of plasticity. Identifying 136 instances of these chemicals among 4892 chemicals within the Comparative Toxicogenomics Database (CTD), we used an *in silico* computational matching approach to systematically assess the ability of these neurotoxicants to dysregulate genes expressed during the peak of the critical period. We identified lead (Pb) as a top hit expected to disrupt critical period plasticity, which we confirmed experimentally in the *in vivo* model of ocular dominance plasticity. This work shows that a systematic, data-driven bioinformatics approach can effectively identify neurotoxicants that pose a risk for childhood brain development.

## Results

### Lead (Pb) identified as a neurotoxicant that disrupts the critical period signature

**Figure 1:**
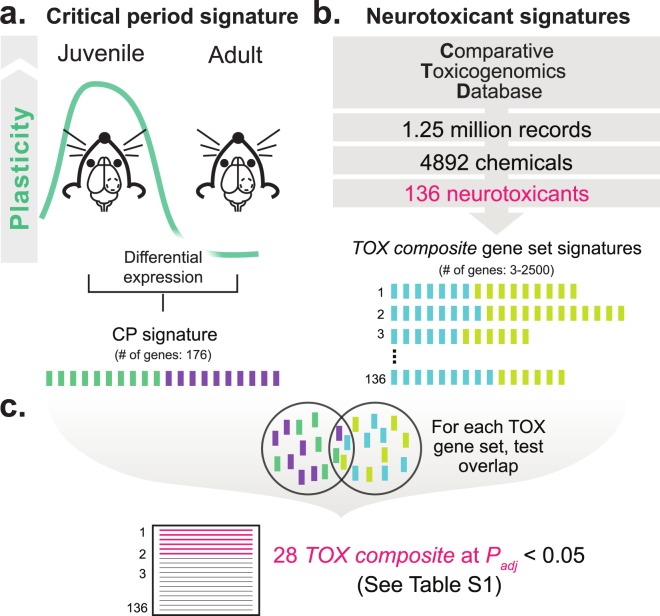
Generation and initial screening of neurotoxicant and critical period transcriptional signatures. (**a**) We generated a critical period signature by differential expression of primary visual cortex (V1) from mouse during the endogenous critical period at postnatal day (P) 26 compared to adult at > P56 using public data (GSE89757) to yield a 176 gene signature. (**b**) From 1.25 million Comparative Toxicogenomics Database (CTD) records across 4892 chemicals with mRNA relationships, we generated 136 neurotoxicant gene sets that included genes both increased or decreased by a given neurotoxicant (*TOX composite* gene set; 3-2419 genes per set). (**c**) We used Hypergeometric tests to assess the likelihood of overlapping genes in the critical period signature with a given *TOX composite* gene set to reduce the search space to 28 neurotoxicants (threshold of *P*_*adj*_ < 0.05) for downstream analysis. See Table S1 for a complete list of the 136 neurotoxicants and related enrichment statistics.

To identify chemicals that potentially disrupt critical period neuroplasticity, we employed an informatics approach to match signatures of environmental chemicals to a signature of a model critical period. As a proof-of-principle, we focused on the subset of 214 chemicals for which previous evidence suggests them as human neurotoxicants^16^. From 1.25 million Comparative Toxicogenomics Database (CTD)^17^ records across 4892 chemicals with mRNA relationships, we identified sufficient data for 136 neurotoxicant signatures (*TOX*). The critical period signature was derived by calculating differential gene expression of the primary visual cortex (V1) in juvenile mice at the peak of the critical period (P26) for ocular dominance plasticity relative to adult mice (Fig. 1a; 176 genes at *P*_adj_ < 0.05; data obtained from: GSE89757^15^). At the peak of the ocular dominance critical period, deprivation of a single eye induces cortical experience-dependent plasticity, a well-characterized critical period model^11^. To match the *TOX* and critical period signatures, we first shrank the search space from the starting 136 neurotoxicants by generating *TOX composite* signatures from transcripts both increased or decreased by a given neurotoxicant (3-2419 genes per signature) (Fig. 1b) and used hypergeometric tests to determine the probability of overlapping genes in a given *TOX composite* signature with all genes in the critical period signature. This analysis identified 28 neurotoxicant signatures that shared genes with the critical period signature regardless of the direction of expression in either the critical period or neurotoxicant gene set (non-directional comparisons considered significant if P_adj_ < 0.05; see Fig. 1c and Table S1).

**Figure 2:**
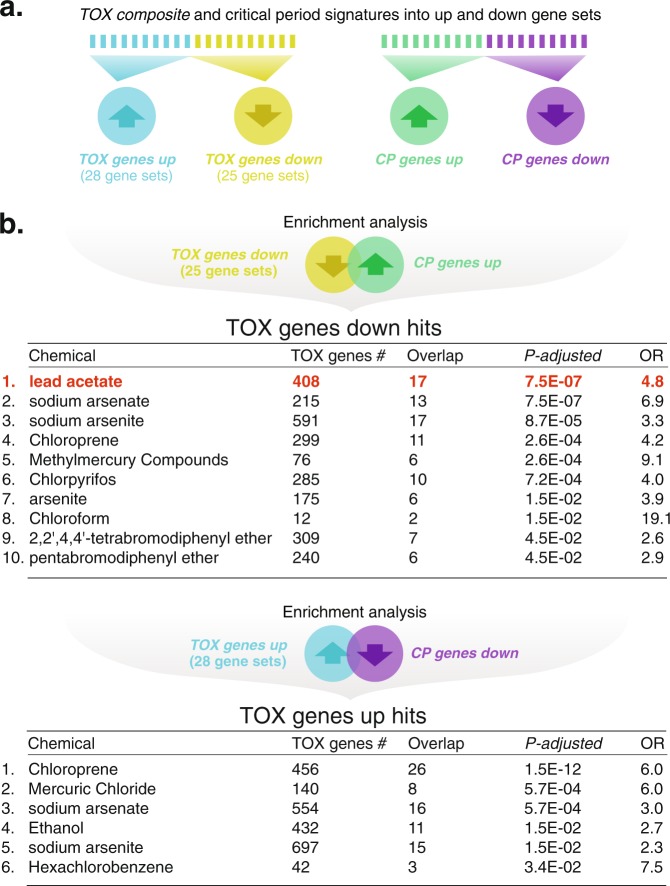
Informatics reveals lead (Pb) as a top neurotoxicant to dysregulate critical period gene expression. (**a**) To facilitate hypothesis testing that the 28 neurotoxicants identified in Fig. 1 disrupt rather than enhance critical period-related gene expression and plasticity, we split the 28 *TOX composite* gene sets into transcripts increased or decreased by a given neurotoxicant (*TOX genes up* and *TOX genes down*) and split the critical period signature into genes increased or decreased in the critical period (*CP genes up* and *CP genes down*). Note: the *TOX genes down* library contains 25 gene sets due to 3 of the gene sets not reaching the minimum size threshold once split. (**b**) Using a directional enrichment analysis by quantifying the overlap of *TOX genes down* with *CP genes up* or *TOX genes up* with *CP genes down* yielded 10 and 6 neurotoxicants expected to reverse critical period gene expression (Hypergeometric tests, threshold *P*_adj_ < 0.05). In both the non-directional (Fig. 1) and directional approaches, lead (Pb) ranked high in its expected ability to dysregulate critical period signature genes (Hypergeometric tests, *non-directional:* OR = 2.4, *P*_adj_ = 1.5 × 10^−05^; *directional:* OR = 4.8, *P*_adj_ = 7.5 × 10^−07^; see also Tables S1 and S2). In the case of ties, results were ordered alphabetically by neurotoxicant name.

To test the hypothesis that these 28 neurotoxicants specifically *reverse* critical period related gene expression, we generated *TOX genes up* (28 gene sets) and *TOX genes down* (25 gene sets) libraries that reflect mRNA transcripts increased or decreased by a given neurotoxicant (note: the *TOX genes down* library contains 25 gene sets due to 3 of the gene sets not reaching the minimum size threshold once split). Similarly, we split the critical period signature into genes increased or decreased in the critical period (CP) signature (*CP genes up* and *CP genes down*) (Fig. 2a). We then performed directional assessments of the overlap of *TOX genes down* and *CP genes up* or *TOX genes up* and *CP genes down* using hypergeometric tests to identify 10 and 6 neurotoxicants that *reverse* critical period gene expression *in silico* (directional comparisons considered significant if *P*_adj_ < 0.05; Fig. 2b,c and Tables S2 and S3) - chemicals that may disrupt functional plasticity.

In both the non-directional and directional analyses, the metallic element lead (Pb) ranked high in its expected ability to dysregulate critical period signature genes (Hypergeometric test, *non-directional:* OR = 2.4, *P*_adj_ = 1.5 × 10^−05^; *directional:* OR = 4.8, *P*_adj_ = 7.5 × 10^−07^; Table S1 and S2 and Fig. 2b). The directional analysis using CTD data found that Pb decreased genes upregulated during the critical period and by qPCR we found after chronic 50 parts-per-million (PPM) Pb exposure in drinking water from P8 that 10 of 16 (63%) had a mean decrease in V1 at the peak of the critical period at P28 and 2 of 16 (12.5%), *Col18a1* and *Mbp*, were significantly lowered (linear models of ΔCTs: −ΔΔCT ≈ log_2_ fold change (FC) = −0.6, *P* = 0.0059, *P*_adj_ = 0.073 and log_2_ FC = −0.51*, P* = 0.0091, *P*_adj_ = 0.073; Pb *N* = 8, Control *N* = 6) (Fig. S1). To further illuminate potential mechanisms, we performed gene set enrichment on genes shared between critical period and neurotoxicant signatures with each of 50 Hallmark^18^ and 5192 Gene Ontology Biological Process gene sets to reveal a common inflammatory signal among Pb and other neurotoxicants (Tables S2 and S3). To test this link in independent data, we compared the overlap of genes most differentially expressed in brain transcriptomes of rodents exposed to Pb[GSE56666^19^] or lipopolysaccharide (LPS) [GSE3253^20^] to find a significant association between Pb and inflammation (Fisher’s Exact test, OR = 1.4, *P* = 0.00012). Gene set enrichment on the 185 shared genes underlying this association found 6 of 7 Hallmark library gene sets related to inflammation were enriched at a *P*_*adj*_ < 0.05 (Fisher’s Exact test, OR = 49.6, *P* = 8.5 × 10^−05^; Table S4). To confirm underlying cytokine signaling, we assessed the similarity of the 185 shared Pb-LPS genes with 96 cytokine and growth factors^21^ to find Interleukin-1 (Il1) as the most strongly associated (Hypergeometric tests, *P*_*adj*_ < 1.3 × 10^−7^; Table S4). Given these enrichments, we assessed by qPCR the impact of chronic juvenile Pb on *Il1β* in V1 to find it was increased (FC = 1.89, *P* = 0.045; Pb *N* = 8, control *N* = 6). These analyses indicate Pb may suppress the critical period signature and suggest inflammation as a potential underlying mechanism.

Given that *TOX* signatures derived from CTD are an aggregation of many studies across diverse tissues, we sought to validate our *in silico* association of Pb and critical period in a brain-specific transcriptional dataset independent of CTD. Adapting a molecular matching algorithm previously validated in brain^15^, we found that Pb at a dose that resulted in blood lead levels (BLLs) most relevant to human childhood exposure (~3 μg/dL)^22^ in rats fed Pb across postnatal development^19^ decreased *CP genes up* (Molecular match score = −3.07, *P* = 0.0039), recapitulating the CTD findings. This effect was specific to brain, as zero of 55 non-brain Pb transcriptional signatures derived from DrugMatrix significantly decreased *CP genes up* after multiple test correction, though there was a skew trending toward Pb signatures decreasing *CP genes up* (i.e. molecular match scores < 0), indicating some fundamental Pb-induced transcriptional mechanisms may be shared between brain and other tissues (D = 0.15, *P* = 0.083, Kolmogorov Smirnov test against a normal distribution) (Table S5). Given the high and brain-specific ranking of Pb by our informatics analyses across independent datasets coupled with the current drinking water crises where elevated Pb poses serious neurodevelopmental consequences to developing children^23^, we focused our study on the role of Pb in disrupting critical period plasticity.

### Pb suppresses critical period experience-dependent plasticity *in vivo*

**Figure 3:**
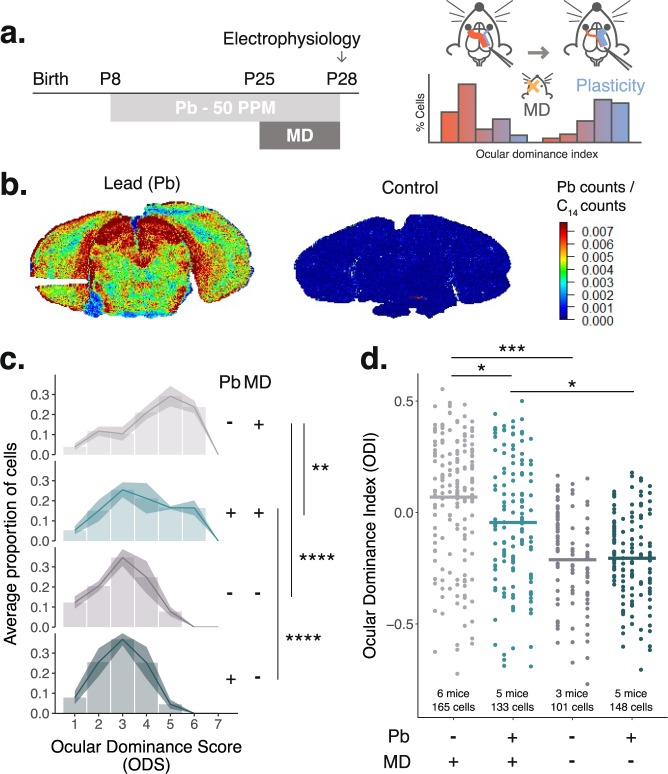
Lead (Pb) suppresses critical period experience-dependent plasticity. (**a**) Mice were administered 50 parts per million (PPM) Pb in drinking water or water alone (control) from P8 through *in vivo* extracellular recordings to assess ocular dominance plasticity at P27-P29 (avg P28) (**b**) Laser ablation-based elemental mapping revealed dramatic Pb accumulation in visual regions including cortical layers of V1 and superior colliculus [Pb *N* = 1, control *N* = 1, both no monocular deprivation (MD)]. (**c**) After 3 days of MD beginning at P24-P26 (avg P25) neurons from control mice (light grey color, 6 mice, 165 cells) exhibited plasticity as quantified at the neuron-level by a shift in their responsivity from the previously deprived eye (contralateral to recording hemisphere) to the nondeprived eye, observable as a right shift in the ocular dominance score (ODS) as compared to control animals who did not receive MD (dark grey color, 3 mice, 101 cells; χ^2^ test of ODS distribution: χ^2^ = 61.3, *P* = 6.6 × 10^−12^). In contrast, V1 neurons from animals who underwent MD and were administered Pb (light teal color, 5 mice, 135 cells) did not exhibit a full ODS shift (Pb MD versus control MD: χ^2^ test of ODS distribution: χ^2^ = 17.1, *P* = 4 × 10^−4^), though some residual plasticity remained (Pb MD versus Pb no MD [dark teal color, 5 mice, 148 cells]: χ^2^ = 42.8, *P* = 4.1 × 10^−8^). (**d**) We carried out an animal-level analysis using a hierarchical linear modeling approach that takes into account within-animal variation (on average, 28.8 neurons were recorded from each mouse) wherein group (Pb or control) and experience (MD or no MD) were assigned as fixed effects and animal was assigned as a random effect to account for repeated neural measurements within each animal. The neuron level ocular dominance index (ODI) was assiged as the continuous outcome variable. After correcting for multiple comparisons using the Holm method, we confirmed plasticity was present in control mice who received MD (light grey color) as quantified by an elevated ODI compared to control animals who did not receive MD (dark grey color) (β = 0.28, *P*_*adj*_ = 0.0002). Mice administered Pb showed significantly reduced plasticity as quantified by a reduction in ODI (Pb MD (light teal color) versus control MD (light grey color): β = −0.11, *P*_*adj*_ = 0.04), but retained some plasticity relative to no MD animals (Pb MD (light teal color) versus Pb no MD (dark teal color): β = 0.16, *P*_*adj*_ = 0.012). Horizontal bars indicate the least squares mean. ****P < 0.0001, ***P ≥ 0.0001 and < 0.001, **P ≥ 0.001 and < 0.01, *P ≥ 0.01 and < 0.05.

To test the hypothesis that Pb exposure disrupts critical period plasticity *in vivo*, we administered 50 PPM Pb in drinking water starting at P8 through to the end of the experiment at a mean age of P28 to model a childhood exposure (Fig. 3a). We sutured one eye to induce experience-dependent plasticity via monocular deprivation (MD) during the peak of the critical period for ocular dominance plasticity at P24-P26. To assess plasticity, three days later (at mean age P28), we removed the sutures from the deprived eye and conducted *in vivo* single-unit recordings of activity-driven changes in the eye preference of single neurons (i.e. ocular dominance) in binocular V1 in response to light^11^ (Fig. 3a). On the day of recording for animals that received MD, blood Pb levels were elevated (2.1 μg/dL vs 0.12 μg/dL in control animals, *t* test: *t* = 5.26, *P* = 0.0062), which reflected the cumulative exposure from the dam’s milk^24^ pre-weaning and from post-weaning consumption of Pb-adulterated water. To assess the spatial distribution of Pb in the brain, we employed laser ablation spectrometry to observe accumulation in visual system regions including V1 and superior colliculus (non-recorded, no MD animals, Pb *N* = 1, control *N* = 1) (Fig. 3b). In a cohort that did not receive MD, we observed no difference on any measure of plasticity between animals that received Pb or control animals that received pure water, as quantified by an animal-level analysis of the contralateral bias index (CBI) and neuron-level analyses of ocular dominance score (ODS) and ocular dominance index (ODI) (Pb CBI = 0.68 ± 0.033 SEM, 5 mice, 148 cells; Control CBI = 0.67 ± 0.032 SEM, 3 mice, 101 cells; animal-level *t* test of CBIs: *P* = 0.93; neuron-level χ^2^ test of ODS counts: χ^2^ = 3.54, *P* = 0.47, neuron-level KS test of ODI distributions: D = 0.06, *P* = 0.64) (Figs 3c and S2a,c). On average, we recorded from 28.8 neurons per animal and the preceding analyses ignored within-animal variation by either averaging multiple within-animal measurements in the case of the animal-level CBI analysis, or by considering the total neurons measured as the *N* in the case of the ODS analysis. Animal-level analyses that take into account within-animal variation using hierarchical linear models (also known as linear mixed models or random effects models) can increase power to detect real differences^25^. Therefore, we assessed using a hierarchical linear model the animal-level differences in ocular dominance index (ODI) between Pb or control animals that did not receive MD to confirm that no difference in plasticity existed (β = −0.0069, *P*_*adj*_ = 0.9) (Fig. 3d). In control animals that received MD, we observed the expected experience-dependent plasticity as quantified by a shift in ocular dominance from the deprived to the nondeprived eye (CBI = 0.45 ± 0.021 SEM, six mice, 165 cells) (Fig. S2b). In contrast, Pb treatment significantly suppressed plasticity as observed by the lack of a shift in ocular dominance quantified by an elevated CBI, a decreased ODI, and an elimination of the right shift in the distribution of ODS from the contralateral to ipsilateral eye (CBI = 0.54 ± 0.039 SEM, five mice, 133 cells; animal-level one-sided *t* test of CBIs: *t* = 1.97, *P* = 0.046; animal-level hierarchical linear model of ODIs: β = −0.11, *P*_*adj*_ = 0.0402; neuron-level χ^2^ test of ODS counts: χ^2^ = 17.13, *P* = 0.004; neuron-level KS test of ODI distributions: D = 0.25, *P* = 9.34 × 10^−05^) (Figs 3c,d and S2b,d). We asked if residual ocular dominance plasticity remained in animals administered chronic Pb to find that some plasticity remains (Pb-MD versus Pb-no MD [CBI = 0.68 ± 0.033, 5 mice, 148 cells], animal-level *t* test of CBIs: *t* = 2.73, *P* = 0.027; animal-level hierarchical linear model: β = 0.16, *P*_*adj*_ = 0.012; neuron-level χ^2^ test of ODS counts: χ^2^ = 42.8, *P* = 4.1 × 10^−08^, neuron-level KS test of ODI distribution: D = X, *P* = 2.11 × 10^−06^) (Fig. 3c,d). Since Pb increases spontaneous neurotransmitter release^26^ and a high spontaneous-to-evoked firing rate ratio is remniscent of an immature cortex^27^, we assessed the rate ratio to find that Pb may increase the rate ratio (Pb-no MD versus control-no MD; neuron-level Kolmogorov Smirnov test: D = 0.16, *P* = 0.039; see Table S6 for summary of firing rate data), though qPCR of GABAergic molecules relevant to cortical maturation were intact (Fig. S3). Together, these experiments validate our integrative bioinformatics screen of neurotoxicants, finding that Pb disrupts critical period plasticity *in vivo* at the peak of the critical period for ocular dominance.

## Discussion

In spite of a lack of systematic screening of the thousands of chemicals in the human environment, evidence has accumulated that at least 214 are neurotoxic to human^16^. Using an integrative informatics approach, we identified dozens of these neurotoxicants as expected to disrupt the critical period for visual cortex ocular dominance plasticity, a well-established model of childhood critical periods of neurodevelopment. We confirmed that a top hit, the metallic element Pb, disrupts *in vivo*, experience-dependent critical period plasticity. This establishes a high-throughput approach to systematically identify neurotoxicants that disrupt critical periods and can be extended to assess other environmental chemicals. Previously, we applied a similar transcriptome-based informatics approach to determine that inflammation is a disease process that disrupts critical period plasticity^15^. Here, we generalize this framework to the identification of neurotoxicants that disrupt plasticity, establishing an informatics approach to match transcriptional signatures as a useful way to identify both disease pathways and neurotoxicants that disrupt critical period plasticity.

Pb was one of dozens of chemicals identified to disrupt a transcriptional signature of critical period neurodevelopment. Perinatal and childhood Pb exposure is consistently associated with decrements in IQ, attention, fine motor control, and mood regulation and *in vitro* and rodent studies have revealed much about the molecular and cellular consequences of such Pb exposure^28^. However, it was previously unknown whether Pb impacts critical period brain plasticity as a mechanism to disrupt childhood neurodevelopment. We show here that Pb at a dose relevant to human exposure during juvenile neurodevelopment disrupts functional, experience-dependent critical period plasticity, by which it may disrupt the neurodevelopmental trajectory to confer risk for neurodevelopmental disorders. Indeed, disruption of critical period plasticity due to juvenile Pb exposure may explain the observation that cases among twins discordant for ASD have increased exposure to Pb during the beginning of the critical period for ocular dominance plasticity [peak elevated Pb at 15–20 weeks of age^2^; peak critical period is ~0.6 to 2 years^13^] and this is associated with reduced IQ later in life^2^. Our study paves the way for further work to explore the hypothesis that Pb disrupts childhood critical periods to confer risk for ASD and other neurodevelopmental disorders.

We postulate a number of potential mechanisms by which Pb disrupts critical period plasticity. First, consistent with previous work^26^ we find that the spontaneous firing rate is increased with Pb administration (see Table S6) and correspondingly the spontaneous-to-evoked firing rate is increased (see Results), suggestive of disruption of inhibition and corresponding delay in the opening of the critical period^27^. However, GABAergic markers relevant to maturation of inhibition of spontaneous firing are intact (see Fig. S3). Moreover, we find some plasticity remains in Pb exposed animals (Pb-MD versus Pb-no MD, see Fig. 3c,d). Together, these data suggest that while Pb may lead to an increased spontaneous-to-evoked ratio, opening of the critical period may be intact and rather suggest a *partial suppression* of critical period plasticity by Pb at the peak of the critical period with increased spontaneous firing as a potential contributing mechanism. Future work should explore known critical period related mechanisms and molecules (i.e. accelerators and brakes) including increased spontaneous firing to tease out the precise nature of the disruption of critical period plasticity by Pb. Second, Pb acts not only on neurons, but upon glia as well. In fact, astrocytes preferentially accumulate Pb^28^. Given that astrocytic secretions of Hevin (also known as Sparcl1) are required for ocular dominance plasticity^29^, Pb exposure may interfere with the physiological role of astrocytes in supporting cortical plasticity. Third, in addition to impacting glia, Pb affects innate immune function including upregulation of inflammatory cytokines including Il1β^30^. Our recent study demonstrated that inflammation by LPS elevates *Il1β* in the brain and disrupts critical period plasticity^15^ and we provide here evidence that suggest Pb may also act through inflammatory pathways to disrupt plasticity. Neurotoxicant gene sets in general were enriched for inflammatory pathways (see Tables S2 and S3) and Pb in particular showed an association with response to exogenous substances and the inflammatory pathway TNF-α via NFkB (see Table S2). Genes differentially expressed in brain by Pb and LPS overlapped and gene set enrichment on these shared genes confirmed inflammatory pathway enrichments, while comparison to cytokine signatures showed Interleukin-1 as the top association (see Table S4), which we confirmed by qPCR after *in vivo* exposure to Pb. Together these analyses are consistent with a role of inflammation downstream of Pb to contribute to disruption of critical period plasticity. Our efforts did not explore whether inflammation is produced peripherally or centrally to impact plasticity. Previously, we administered intraperitoneal a dose of LPS reported not to cross the blood brain barrier and this suppressed plasticity at the peak of the critical period for ocular dominance^15^, indicating that peripherally-generated inflammation is sufficient to suppress ocular dominance plasticity. While peripheral inflammation may be the result of an innate immune response to the exogenous substance Pb, given the association between microbiome and inflammatory disease^31^, an alternative hypothesis is that disruption of the microbiome by Pb^32^ could lead to increased inflammation mediated by gut dysbiosis. Administering a Pb-chelating chemical or anti-inflammatory compound that cannot cross the blood brain barrier co-current with Pb administration may help to tease out central versus peripheral impact of Pb. Collectively, Pb may simultaneously impact multiple cell-types including neurons, astrocytes, and peripheral cells to activate both inflammatory and other pathways to accumulate in the disruption of critical period plasticity.

This work shows that a systematic, data-driven, transcriptome-based approach can effectively identify neurotoxicants of critical period plasticity. A limitation of this study is that there was only sufficient data within the CTD for 136 of 214 known neurotoxicants to reliably assess their impact on critical period plasticity. Moreover, the use of publically available data relies on the investigation of chemicals that have already been deemed interesting in the past. This bias could be corrected by systematic assessment of all widely-used chemicals on the human transcriptome using cell line assays as has been done with therapeutic small molecules^33^. Similarly, in this study we limited ourselves to a single critical period signature that reflected endogenous gene expression relevant to the visual cortex at the peak of the critical period for ocular dominance. Future work can build on this by using additional genetic and environmental (i.e. running-induced plasticity, dark-rearing) models of critical period-related plasticity^5^ to screen for neurotoxicants that impact specific aspects of critical period plasticity as well as point toward underlying mechanisms.

Future studies can build upon this by extending beyond the 136 neurotoxicants surveyed here to the 4892 chemicals in the CTD for which sufficient transcriptional data is available. To increase the fidelity of *in silico* transcriptional screening, ongoing efforts to systematically profile expression across approximately 1500 genes relevant to toxicology (i.e. the S1500 platform) induced by tens of thousands of chemicals by the U.S. multiagency collaborative, “Toxicology in the 21st Century” (Tox21 program), are a hopeful boon to high-throughput screening of environmental chemicals^8^. To address the critical need of screening environmental chemicals for their impact on neurodevelopment, S1500 efforts must be extended to mouse primary and human induced pluripotent stem cell (iPSC)-derived cell lines to complement ongoing efforts using RNA-sequencing^9^. Similarly, it is important to extend existing efforts to build models of toxicity using human lymphoblastoid cell lines^34^ to human iPSC-derived neurons towards prediction of neurodevelopmental effects. Another important future direction is to consider mixtures of chemicals^35^ and the interaction between chemical and environmental exposures, starting with pairs of exposures such as Pb and stress^36^ and moving to consider the totality of environmental exposures - the exposome^37^.

In summary, we demonstrate here an integrative bioinformatics approach to systematically identify neurotoxicants that disrupt *in vivo* critical period neuroplasticity. This approach may be immediately extended to efficiently screen other environmental chemicals as well as generalized to other brain phenotypes to identify chemicals that impact diverse aspects of brain development. Given the recent child health crises in Flint, MI, USA, and elsewhere in the world, with elevated levels of Pb and other chemicals in public drinking water supplies^23^, implementing high-throughput approaches to identify dangers to childhood neurodevelopment is an important step in safeguarding child health.

## Materials and Methods

### Research objectives

The objective of this study was to determine if an integrative bioinformatics approach could be applied to systematically identify environmental chemicals that disrupt critical periods of neuroplasticity as tested subsequently in the *in vivo* ocular dominance model of critical period plasticity. The study began with a systematic, hypothesis-generation step (the computational step that includes Figs 1 and 2) where we prioritized hypotheses about what neurotoxicants may disrupt plasticity. Prior to the outcome of these analyses, we hypothesized that top neurotoxicant hits would reverse gene expression important in the critical period. From these analyses, we hypothesized a top ranked neurotoxicant lead (referred to in this paper by its chemical element symbol Pb) would suppress functional plasticity. When we found this to be the case, we further hypothesized that the spontaneous-to-evoked firing rate ratio would increase. Both the informatics approach and validation model were specified beforehand and a single top chemical, Pb, was chosen to test the approach, the results of which are reported here.

### Animals

Male C57Bl6 mice (Charles River Laboratories) were group housed under a standard 12 h light/dark cycle (lights on at 7:00 A.M., lights off at 7:00 P.M.) with constant temperature (23 °C) and ad libitum access to food and water. The Institutional Animal Care and Use Committee at the Icahn School of Medicine at Mount Sinai approved all procedures involving animals, and were carried out in accordance with the National Institute of Health guide for the Care and Use of Animals.

### Experimental design

We performed a controlled laboratory experiment where mice were given either Pb in drinking water or pure water alone chronically during the juvenile period and up through the endpoint, either qPCR of brain cortex, brain sectioning, or electrophysiology of brain cortex.

### Randomization

Pups were randomly chosen to be in the Pb or pure water groups and cross-fostered, when possible.

### Blinding

The experimenter performing the ocular dominance plasticity assay was blinded as to the conditions (the animal was delivered to the technician by another author who was not otherwise involved in the experimental assay nor the subsequent statistical analysis, but was involved in the assessment of the quality of the raw data and inclusion/exclusion).

### Rules for stopping data collection and data inclusion/exclusion criteria

We stopped data collection when we reached the sample size estimated by apriori power calculation. Inclusion and exclusion criteria for the ocular dominance assay were previously established^15^ and applied here (see below section *In vivo electrophysiology* for a restatement of these criteria). Outliers were not assessed nor excluded.

### Selection of endpoints

Two endpoints were selected. Primary endpoint: the animal-level contralateral bias index and animal-level hierarchical linear model of ocular dominance index. Secondary endpoint: the neuron-level ocular dominance score and neuron-level ocular dominance index.

### Replicates

A minimum of 3 biological replicates were included in all experiments, whether completed in house or re-analyzed from public data. Ocular dominance assay and qPCR experiments were performed once. For qPCR, three technical replicates were always included in the assay and averaged.

### Critical period signature

Juvenile critical period signature was generated from publically available juvenile mice data, GSE89757^15^). Briefly, we used Limma^38^ to quantile normalized raw microrray probe-level data and RankProd^39^ to compute rank-based differential expression of mouse genes between juvenile mice at postnatal day (P) 26 and adult (>P56) C57Bl6 mice (n = 3 each group) primary visual cortex (V1), which we mapped to orthologous human genes using the Mouse Genome Informatics homology reference to yield 176 genes. Differentially expressed genes were split into those increased and decreased during the critical period (*CP genes up* and *CP genes down*). To enable gene set enrichment analyses via hypergeometric tests the critical period transcriptome was computed. Probe-level data from above microarray dataset was background corrected, quantile normalized, and log2 transformed with Limma and then collapsed to human gene orthologs using the Mouse Genome Informatics homology reference (maximum mean intensity value was retained in cases of multiple probes mapping to the same gene) to yield a 9657 gene transcriptome.

### Neurotoxicant signatures

Neurotoxicant signatures were derived from Comparative Toxicogenomics Database (CTD) data. From 1.25 million CTD relationships between chemicals and 33 biological substrates (i.e. protein, DNA, mRNA, etc), chemicals with mRNA relationships were retained to yield 4892 chemicals. Of these chemicals, 195 were shared with a list of 214 unique human neurotoxicants identified by Landrigan and Grandjean^16^. For these 195 neurotoxicants, composite gene set signatures (genes increased AND decreased by a given neurotoxicant) were generated to yield a library of 136 gene sets (*TOX composite*) after filtering for gene set size (min = 3, max = 2500 genes). For subsequent analyses based on neurotoxicants with significant overlaps between a given composite gene set and the critical period signature, we generated *TOX genes up* (28 gene sets) and *TOX genes down* (25 gene sets) libraries, which reflect genes increased or decreased by a given neurotoxicant. Gene set size varied between *TOX genes up* and *TOX genes down* gene sets due to again filtering by size (min = 3, max = 2500 genes) after splitting the *TOX composite* gene sets.

### Enrichment analyses

To determine statistical enrichments of neurotoxicant gene set signatures and critical period signatures, the critical period signature was matched to neurotoxicant signatures using hypergeometric tests. This test aims to identify the probability of genes in a neurotoxicant signature overlapping with genes in the critical period signature, given the background of all genes potentially relevant to both critical period and neurotoxicants. This background was computed by taking the intersection of the critical period transcriptome and genes associated with any of the 4892 chemicals with mRNA relationships in the CTD mapped to human orthologous Entrez gene ids (9641 genes). Hypergeometric tests were computed between given *TOX composite* gene sets and all genes differentially expressed in the critical period to reduce the search space to 28 neurotoxicants that shared genes with the critical period regardless of the direction of expression in either the critical period or neurotoxicant signature. Next, to determine if a given neurotoxicant reversed critical period gene expression, the overlap of gene sets of the *TOX genes down* and *CP genes up* or the *TOX genes up* and *CP genes down* were computed using hypergeometric tests. To better understand the potential biological role of neurotoxicant genes that were shared with the critical period signature, we calculated gene set enrichments using hypergeometric tests to assess the probability of the overlap of shared neurotoxicant-critical period genes with each of 50 Hallmark gene sets^18^ and 5192 Gene Ontology Biological Process gene sets [using the Enrichr build^40^]. In all cases, a threshold of *P*_adj_ < 0.05 was set to consider enrichments significant.

### Pb and lipopolysaccharide transcriptome signatures

Pb and lipopolysaccharide (LPS) transcriptome-wide signatures were curated from Gene Expression Omnibus (GEO) and included a dataset of juvenile rat hippocampus chronically exposed to Pb in chow (GSE56666), 55 instances of Pb exposure to rats from non-brain tissues exposed to various doses of Pb by oral gavage, for various periods of time (GSE59927), and whole brain homogenate 4 hours after low dose (LPS) injected intraperitoneal (GSE3253). GSE56666 included data for males and females across 3 species; given our test animals were male we used only male data in generation of the signature and considered all samples together (did not separate by strain). Data was normalized and differential transcriptomes computed as previously published^41^. Briefly, raw data were downloaded from GEO (for DrugMatrix, this was facilitated by metadata derived by Ma’ayan Lab: http://amp.pharm.mssm.edu/CREEDS/#downloads^42^), normalized by a rank-based approach (RankNorm) by ordering the expression values from highest to lowest and applying a rank where the highest expressed gene was N = total number of genes and lowest ranked gene was 1. Ranks were normalized to the range 0–1 inclusive by dividing all ranks by N. In cases where multiple probes mapped to the same gene the gene with highest average rank across samples was retained. Differential expression across the entire transcriptome was computed as the difference in rank between case and control (SubDiff), yielding a differential expression value ranging from −1 to +1. Code to generate these signatures was based on a microarray analysis pipeline built in-house^43^. To determine if genes shared by Pb and LPS exposure significantly overlapped, we first identified genes differentially expressed as those with a SubDiff Z-score of > 1.5 or <−1.5 (Pb 1125 genes, LPS 1485 genes). We then calculated the probability of the 185 shared genes relative to a background of all 11,582 genes commonly expressed on the Pb or LPS microarrays using a Fisher’s Exact test (all genes were mapped to orthologous human Entrez gene ids). Using this same background, we employed hypergeometric tests to identify 50 Hallmark gene sets and 96 Library of Integrated Network-based Cellular Signatures (LINCS) ligand gene sets^21^ (gene expression response of cell lines to cytokine and growth factors, range of signatures 273–327 genes - generated by combining genes identified as increased and decreased by a given ligand in the Enrichr build 2017^40^) that were associated to the shared 185 Pb-LPS genes and considered any Hallmark or LINCS ligand gene sets significant if the *P*_*adj*_ < 0.05.

### Molecular matching

A molecular match score was adapted from^15^ to quantify the ability of a given Pb signature to decrease *CP genes up*. Briefly, summing the SubDiff expression values for genes in a given transcriptome signature that are present in the *CP genes up* signature yielded the molecular match score (M): a summary measure of concordance between critical period signature and Pb transcriptome gene expression. Low molecular match scores (<0) indicate that a given Pb signature decreases genes upregulated in the critical period and high scores (>0) indicate the Pb signature increases these genes. Therefore, we would hypothesize a low molecular match score indicates that critical period gene expression and plasticity may be suppressed *in vivo*. To compare match scores (M) across transcriptome signatures (e.g. between instances in DrugMatrix), we normalized each M using $\frac{{\rm{M}}-{\bar{{\rm{M}}}}_{perm}}{\sqrt{\frac{{\sum }_{i=1}^{n}{({{\rm{M}}}_{per{m}_{i}}-{\bar{{\rm{M}}}}_{perm})}^{2}}{n-1}}}$ where ${\bar{{\rm{M}}}}_{perm}$ is the mean of *n* = 10,000 permutations of scores generated by shuffling the gene labels and recalculating M. *P* values for M were estimated from ${\bar{{\rm{M}}}}_{perm}$ using the Generalized Pareto Distribution^44^ on *n* permutations. M is computed similar to the approach by Zhang and Gant^45^.

### Substances

1.144 g of Pb (lead (II) acetate trihydrate; Sigma-Aldrich, Cat# 467863) was dissolved in 25 mL of Milli-Q ultrapure deionized water (dH20) to yield a stock solution of 45.77 mg/ml. 600ul of the stock solution was diluted to 300 ml with dH20 to yield a 50 parts-per-million (PPM) working solution.

### Lead (Pb) experimental design

Animals were received on P7, acclimatized for one day, and then divided into groups receiving 50 PPM Pb in dH20 or dH20 alone as drinking water until P27–29 (average P28) when qPCR, sectioning, or electrophysiological recordings took place.

### *In vivo* electrophysiology

Under light anesthesia, the eye contralateral to the recording site of experiment-naive P24-27 mice was sutured (monocular deprivation; MD) under light isoflurane and three days later, single-unit electrophysiological recordings were taken in binocular zone of V1 in response to visual stimuli presented to each eye separately^11,15^ (*N* = 5 Pb, *N* = 6 control). Animals were weaned at MD. A separate cohort of animals were recorded without MD to assess baseline activity (*N* = 5 Pb, *N* = 3 control). Animals were anesthetized with nembutal/chlorprothixene anesthesia and atropine and dexamethasone were injected subcutaneous to reduce salivary secretion and brain swelling, respectively, during recording. Visual responses evoked by a high contrast single bar generated by ViSaGe system (Cambridge Research System) were recorded using a 16 channel probe. The exclusion criteria to discard recordings were failed MD (identified by opening of sutured eye) or poor recording quality (<10 cells/mouse, <3 penetrations/mouse, or inability to identify both monocular zone and secondary visual cortex). To analyze the electrophysiology data, normalized ocular dominance index (ODI) of single neurons was computed by a custom MATLAB code via peristimulus time histogram analysis of peak to baseline spiking activity in response to each eye: 〈[Peak(ipsi)-baseline (ipsi)]–[Peak (contra)-baseline(contra)]〉/〈[Peak (ipsi) – base line(ipsi)] + [Peak(contra)-baseline(contra)]〉, which produces a range of [−1, +1] where −1 is a completely contra-dominated cell and +1 is a completely ipsi-dominated cell. ODI is linearly transformed by assigning [−1.0, −0.5) = **1**, [−0.5, −0.3) = **2**, [−0.3, −0.1) = **3**, [−0.1, +0.1] = **4**, (+0.1, +0.3] = **5**, (+0.3, +0.5] = **6**, (+0.5, +1.0] = **7** to produce the ocular dominance score (ODS). Finally, the contralateral bias index (CBI), a monocular weighted, animal-level summary statistic, is computed from the ODS: [(n**1**-n**7**) + 2/3(n**2**-n**6**) + 1/3(n**3**-n**5**) + N]/2 N, where N = total number of cells and n**x** = number of cells corresponding to an ODS of **x**. Thus, a CBI of 0.7 is contra-dominant and a CBI of 0.4 is ipsi-dominant. For comparison of ocular dominance, we plotted CBI of single animals in their respective groups and statistically compared between groups via *t* tests. Additionally, we computed the average proportion of ODS counts for a given bin (integers 1 through 7) across animals, plotted these average proportions as histograms and included the standard error of the mean (SEM) as a shaded region around the mean, and statistically compared the raw ODS counts between groups via the non-parametric Chi-squared test. Finally, we plotted each ODI for each neuron for each animal in each group to show the variation within and between groups and used hierarchical linear modeling (also known as linear mixed modeling or random effects modeling) to statistically compare the groups while accounting for both within and between animal variation^25^ using the R packages lme4 (v. 1.1.12), LmerTest (v. 2.0.32), and lsmeans (v. 2.25). Experience (MD or no MD) and group (Pb or control) were modeled as fixed effects and animal was modeled as a random effect (animals were considered an independent and random sample from the population and neural measurements within an animal as nested, repeat measures). We assigned the neuron-level ODI as the outcome variable as it is the only continuous variable that encodes ocular dominance of a given neuron. The Holm method was used to correct comparisons of multiple contrasts and those with *P*_*adj*_ < 0.05 were considered significant. Immediately after recording, trunk blood and non-recorded V1 were collected for further analysis. We estimated sample size apriori via power analysis assuming the effect size and standard deviation from previous studies at *N* = 5–6, which we obtained in the study.

### Quantitative PCR (qPCR)

Mice not subjected to MD whose brains were not recorded from (*N* = 3 Pb, *N* = 3 control) or were recorded from (*N* = 5 Pb, *N* = 3 control) were anesthetized under 3.0% isoflurane or nembutal/chlorprothixene anesthesia, rapidly decapitated, and bilateral V1 dissected under RNAse-free conditions, briefly rinsed in sterile saline (0.9% NaCl), frozen on dry ice, and stored at −80 °C until further processing. Total RNA was extracted from unilateral V1 using the RNeasy Lipid Tissue Mini Kit (Qiagen) and stored at −80 °C. RNA yields ranged from 1.5 to 10.5 μg/sample with a mean of 5.6 μg. Total V1 RNA was converted to cDNA using a High Capacity cDNA Reverse Transcription Kit (Life Technologies). A FX^P^ Biomek Liquid Handler or manual approach was used to plate cDNA, Taqman Master Mix II, and Taqman probes (*Marcksl1*: Mm00456784_m1, *JunB*: Mm04243546_s1, *Col22a1*: Mm01195058_m1, *Tnc*: Mm00495662_m1, *Kank1*: Mm00619389_m1, *Col18a1*: Mm00487131_m1, *Fermt1*: Mm01270148_m1, *Ppapdc1a*: Mm01276440_m1, *Fos*: Mm00487425_m1, *Cd93*: Mm00440239_g1, *Adam19*: Mm01286004_m1, *Gapdh*: Mm99999915_g1, *Eif2ak1*: Mm01202300_m1, *Arc*: Mm01204954_g1, *Egr2*: Mm00456650_m1, *Npas4*: Mm01227866_g1, *Mbp*: Mm01262035_m1, *Gad1* (also known as *Gad67*): Mm04207432_g1, *Gad2* (also known as *Gad65*): Mm00484623_m1, *Pvalb*: Mm00443100_m1, *Vgat* (also known as *Slc31a1*): Mm00494138_m1, *Gabra1*: Mm00439046_m1, *Gphn*: Mm00556895_m1, *Il1β*: Mm00434228_m1). qPCR plate reading was performed by the Mount Sinai Quantitative PCR core facility. Using SDS 2.4, raw fluorescence signals were normalized to baseline, quality of amplification was assessed, and cycles to threshold (CT) were calculated. *Fermt1* data was discarded due to poor amplification. ΔCT were calculated using as reference the geometric mean of two housekeeping genes, *Gapdh* and *Eif2ak1*. Quantification of the log2 fold change between Pb and control conditions for a given gene was derived via linear regression with ΔCT as the outcome variable and recording status (recorded or not recorded), group (Pb or control) and an interaction term as the independent variables. In this scenario, the coefficient for group effect is equivalent to the ΔΔCT and we took the negative of this coefficient to yield the −ΔΔCT (equivalent to a log2 fold change). This approach is reasonable under the assumptions of the linear model given the approximately normal distribution of ΔCTs.

### Pb tissue analyses

*Brain:* Mice not subjected to MD (*N* = 1 per group) were lightly anesthetized via 3.0% isoflurane, decapitated, and whole brain was rapidly frozen in optimal cutting tempature (OCT) compound on dry ice and stored at −80 °C. Brains were coronal sectioned at 10 μm thick using a cryostat, placed directly on glass slides, briefly air dried, and stored at −20 °C. Brain sections were analyzed for metal concentrations using laser ablation-inductively coupled plasma-mass spectrometry (LA-ICP-MS). The surface of the tissue section was rastered with a laser ablation unit (NWR 193 nm) and the ejected material ionized in a mass spectrometer (QQQ 8800 Agilent ICP-MS). Helium is used as a carrier gas from the laser ablation cell and mixed with argon via Y-piece before introduction to the ICP-MS. The system was tuned daily using National Institute of Standards and Technology SRM 612 (trace elements in glass) to monitor sensitivity (maximum analyte ion counts), oxide formation (232Th16O+/232Th+, <0.3%) and fractionation (232Th+/238U+, 100 ± 5%). *Blood:* Trunk blood was collected immediately after recording in MD (Pb *N* = 5, control *N* = 6) and no-MD (Pb *N* = 5, control *N* = 3) or at sacrifice in no-MD animals who were not recorded (Pb *N* = 3, control *N* = 3), and no-MD non-recorded animals whose brains were sectioned to analyze the cerebral Pb distribution and quantity (Pb *N* = 1, control *N* = 1). Blood was subjected to in-solution ICP-MS to determine blood Pb levels.

### Statistical Analysis

Statistical analyses were completed in the R programming language (v 3.2.2). In all cases of multiple hypothesis testing, resulting *P* values were corrected using the False Discovery Rate (FDR) approach^46^ and is referred to as *P*-adjusted (*P*_adj_) throughout the paper. All tests were 2-sided, except where otherwise specified (for comparing the CBI in animals that received MD, we used a one-sided *t* test since we hypothesized *a priori* that Pb would specifically increase the CBI in Pb versus control). Hypergeometric tests were computed via the HTSanalyzeR R package^47^ and Fisher’s Exact tests via the base R function.

## Electronic supplementary material

FigS1–3

TableS1

TableS2

TableS3

TableS4

TableS5

TableS6

## Data Availability

The microarray gene expression data analyzed in this study is publically available on the Gene Expression Omnibus (accession numbers stated in relevant Methods section). Ocular dominance and qPCR data available from the corresponding author on request.
