# Supplementary material for: Integrative bioinformatics identifies postnatal lead (Pb) exposure disrupts developmental cortical plasticity
Source: Sci Rep. 2018 Nov 6;8:16388. doi: 10.1038/s41598-018-34592-4 (PMC6219596; doi:10.1038/s41598-018-34592-4)
Supplement: Supplementary file 1 — FigS1–3 [file 41598_2018_34592_MOESM1_ESM.pdf]

Title

Full title. Integrative bioinformatics identifies postnatal lead (Pb) exposure disrupts developmental cortical plasticity

Short title. Lead (Pb) disrupts plasticity

Authors

Milo R Smith[1-4,7-10] Priscilla Yevoo [1,3-4,7,10], Masato Sadahiro[1,3-4,7,8,10], Christine Austin[5], Chitra Amarasiriwardena[5], Mahmoud Awawda[5], Manish Arora[5,6], Joel T Dudley[2,9]*, Hirofumi Morishita[1,3-4,7,8,10]*

Affiliations

[1]Department of Neuroscience.

[2]Department of Genetics and Genomic Sciences.

[3]Department of Psychiatry.

[4]Departmnt of Ophthalmology.

[5]Department of Environmental Medicine & Public Health.

[6]Department of Dentistry.

[7]Mindich Child Health and Development Institute.

[8]Graduate School of Biomedical Sciences.

[9]Institute for Next Generation Healthcare.

[10]Friedman Brain Institute.

Icahn School of Medicine at Mount Sinai, 1 Gustave L Levy Place, New York NY, 10029.

*hirofumi.morishita@mssm.edu

*joel.dudley@mssm.edu

Supplementary Figures

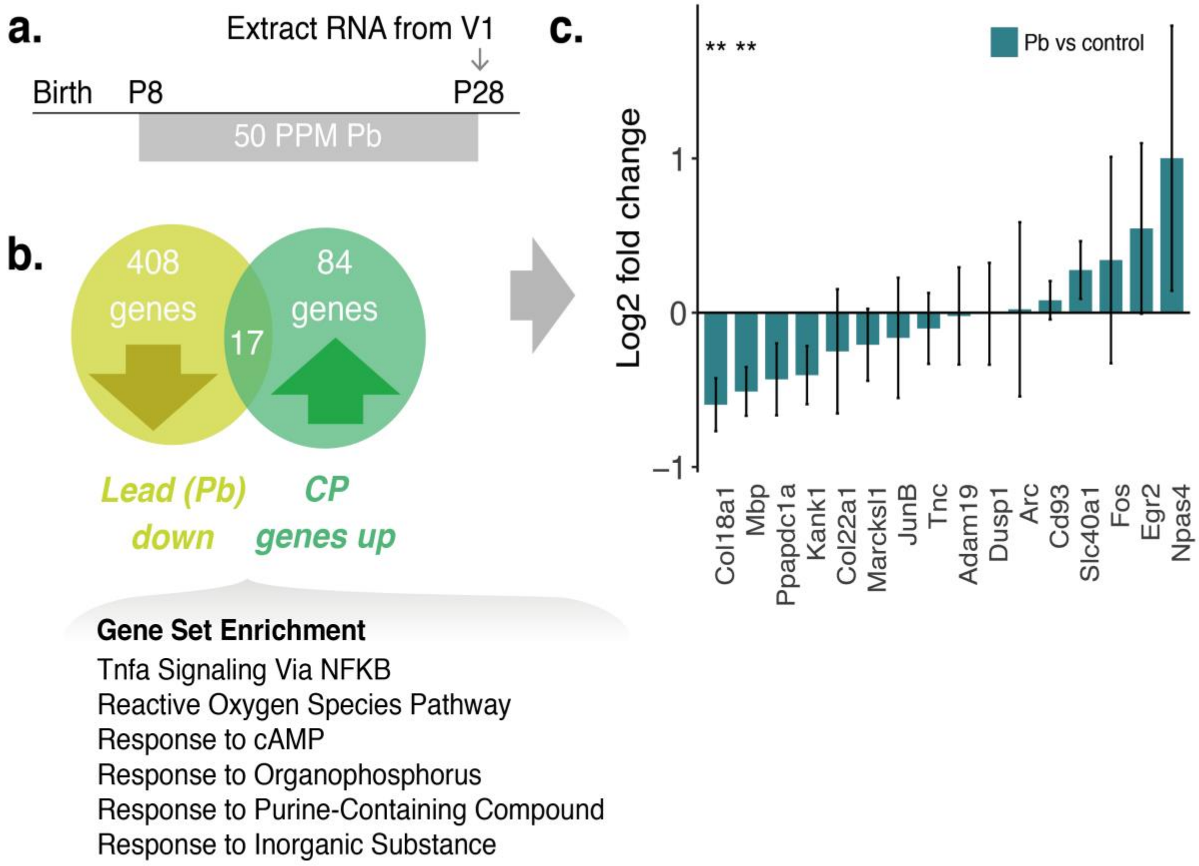

Supplementary Figure 1. Juvenile lead (Pb) reverses critical period gene expression. (**a**) We administered 50 PPM Pb in drinking water or water alone starting at P8 to model a childhood exposure. (**b**) Taking the transcripts upregulated in the critical period expected to be decreased by Pb from our informatics screen, we identified 17 genes, which we subjected to qPCR from V1 of Pb (N = 8) and control treated animals (N = 6) at P28. Gene set enrichments for these 17 genes included inflammatory and response to exogenous substance pathways (those included with $P_{adj} < 0.05$). (**c**) After removing one gene (*Fermt1*) whose probe did not allow proper amplification, we found that as expected 10 of 16 (63%) had a mean $\log_2$ fold change decrease after Pb exposure and 2 of 16 (12.5%), Col18a1 and Mbp, were significant (linear models of ΔCTs: -ΔΔCT ≈ $\log_2$ fold change (FC) = -0.6, P = 0.0059, P_{adj} = 0.073 and $\log_2$ FC = -0.51, P = 0.0091, P_{adj} = 0.073). (Two additional genes, Ppapdc1a and Kank1, were near the nominal significance threshold of P = 0.05). ** $P \geq 0.001$ and ≤ 0.01). P values adjusted for multiple tests using the Benjamini and Hochberg (1995) approach (P_{adj}).

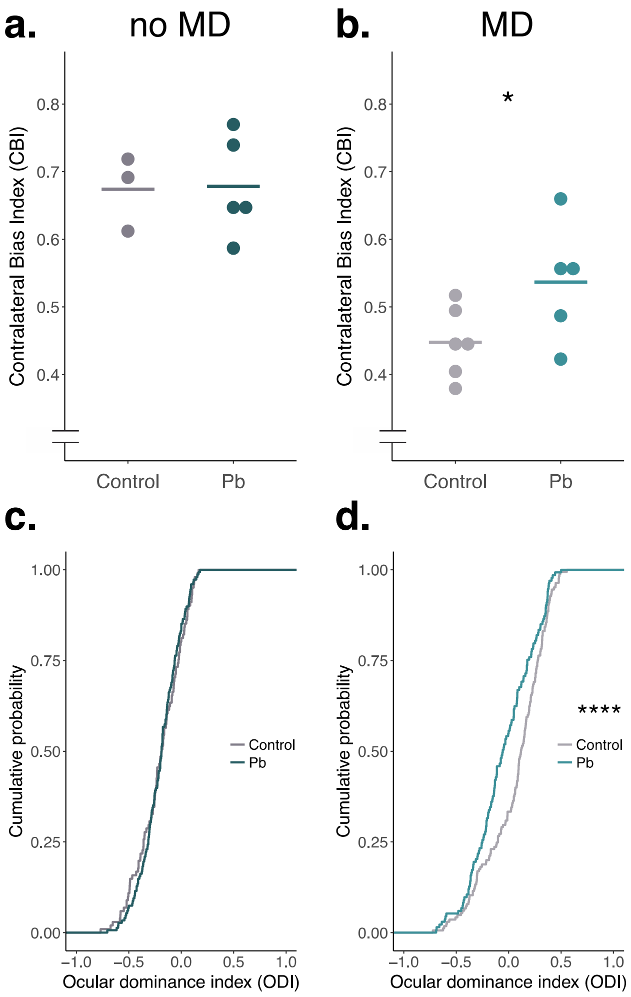

Supplementary Figure 2. Traditional animal-level contralateral bias index (CBI) and neuron-level ocular dominance index (ODI) analyses indicate lead (Pb) suppresses critical period experience-dependent plasticity (related to Figure 3). Mice administered 50 PPM Pb in drinking water or water alone (control) from P8 through in vivo extracellular recordings to assess ocular dominance plasticity at P27-P29 (avg P28). (**a**) Pb and control animals that did not receive monocular deprivation (no MD) did not differ in their plasticity as quantified by CBI (Control no MD: dark grey color, *N* = 3 mice, CBI = 0.68, Pb no MD: dark teal color, *N* = 5 mice, *t* test of CBIs: *P* = 0.93). (**b**) After 3 days of MD, Pb decreased plasticity as quantified by an increased CBI in Pb versus control (control MD: light grey color, *N* = 6 mice, CBI = 0.45 ± 0.021 SEM; Pb MD: light teal color, *N* = 5 mice, CBI = 0.54 ± 0.039 SEM; one-sided *t* test of CBIs: *P* = 0.046. One-sided *t* test chosen due to apriori hypothesis that Pb would decrease plasticity (increase CBI)). (**c**) Cumulative distributions of ODI are not different for Pb exposed and control mice with no MD (KS test of ODI distributions: D = 0.06, *P* = 0.64) (**d**) No right shift in ODI distribution after MD in mice exposed to Pb relative to control mice (KS test of ODI distributions: D = 0.25, *P* = 9.34 x 10^{-05}). Horizontal bars for the CBI plot indicate the mean. **** *P* < 0.0001, * P ≥ 0.01 and ≤ 0.05.

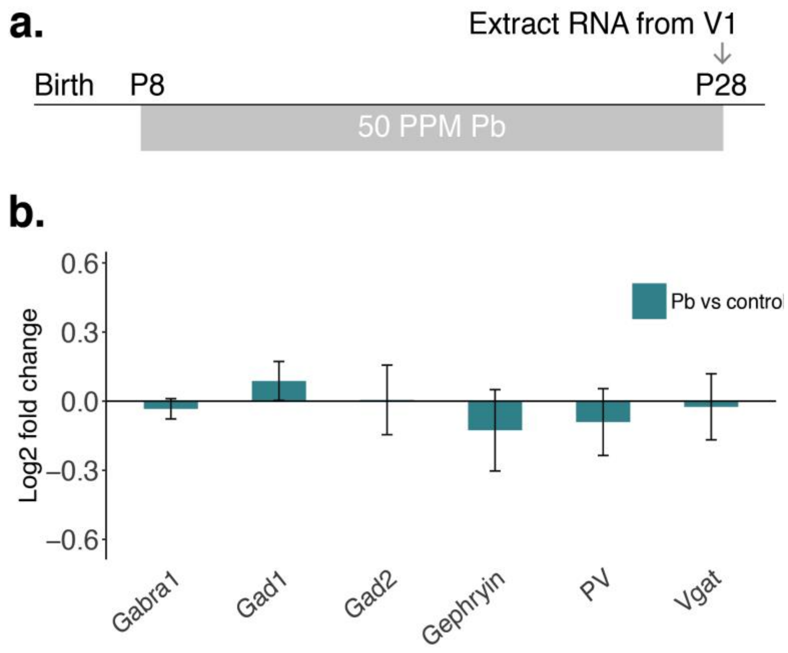

Supplementary Figure 3. Juvenile lead (Pb) does not alter GABAergic genes related to maturation of inhibition. (**a**) We administered 50 PPM Pb in drinking water or water alone starting at P8 to model a childhood exposure. (**b**) We assessed via qPCR primaryvisual cortex (V1) for the levels of transcripts relevant to the maturation of inhibitory neurons and did not identify differences between Pb and control (only samples from no MD non recorded animals used: Pb $N = 3$, control $N = 3$).
